# High-Energy Phosphates and Ischemic Heart Disease: From Bench to Bedside

**DOI:** 10.3389/fcvm.2021.675608

**Published:** 2021-07-28

**Authors:** Hao Yi-Dan, Zhao Ying-Xin, Yang Shi-Wei, Zhou Yu-Jie

**Affiliations:** The Key Laboratory of Remodeling-Related Cardiovascular Disease, Ministry of Education, Beijing Institute of Heart, Lung and Blood Vessel Disease, Beijing Anzhen Hospital, Capital Medical University, Beijing, China

**Keywords:** high-energy phosphates, creatine phosphate, energy metabolism, ischemic heart disease, cardioprotection

## Abstract

The purpose of this review is to bridge the gap between clinical and basic research through providing a comprehensive and concise description of the cellular and molecular aspects of cardioprotective mechanisms and a critical evaluation of the clinical evidence of high-energy phosphates (HEPs) in ischemic heart disease (IHD). According to the well-documented physiological, pathophysiological and pharmacological properties of HEPs, exogenous creatine phosphate (CrP) may be considered as an ideal metabolic regulator. It plays cardioprotection roles from upstream to downstream of myocardial ischemia through multiple complex mechanisms, including but not limited to replenishment of cellular energy. Although exogenous CrP administration has not been shown to improve long-term survival, the beneficial effects on multiple secondary but important outcomes and short-term survival are concordant with its pathophysiological and pharmacological effects. There is urgent need for high-quality multicentre RCTs to confirm long-term survival improvement in the future.

## Introduction

The heart is more than a hemodynamic pump. It is also an organ that needs energy from metabolism (1). In fact, altered cardiac metabolism is the primary and upstream pathophysiologic manifestation of myocardial ischemia in humans (2). After coronary blood flow blockage, energy metabolism disorder occurs within a few seconds, followed by mechanical, electrophysiological and structural abnormalities of the myocardium. To date, standard treatments for ischemic heart disease (IHD), including revascularization (thrombolysis, percutaneous coronary intervention, and coronary artery bypass grafting), antithrombotic therapy (antiplatelet and anticoagulant agents), stabilization/reversal of atherosclerosis progression (control of atherosclerotic risk factors), and inhibition of myocardial remodeling (sympathetic and renin-angiotensin-aldosterone system inhibitors), focus on coronary anatomy and on the results of changes in myocardial metabolism rather than on the metabolic changes themselves (2–8). In addition, almost all of the above treatments exert cardioprotection by directly or indirectly affecting heart rate, blood pressure or myocardial perfusion. In contrast, myocardial energy metabolic therapy (MEMT) plays a protective role by regulating the energy synthesis and utilization of myocardial cells without significant impacts on heart rate, blood pressure and perfusion (9, 10). Because of residual cardiovascular risk, MEMT is promisingly emerging as an upstream treatment for IHD (11).

Since the discovery of creatine phosphate (CrP) in 1927 (12) and adenosine triphosphate (ATP) in 1929 (13), the biochemical, physiological, and pharmacological properties of high-energy phosphates (HEPs) have been gradually uncovered. Unlike the single metabolic process of glucose, free fatty acids or amino acids, the pathways and regulations of HEPs biosynthesis and degradation are involved in all metabolic substrates. Moreover, due to the production and consumption of HEPs in different cells and subcellular organelles, the transmembrane transport of HEPs is also a complex process requiring the assistance of many special transporters and catalytic enzymes (14). Therefore, although HEPs have been known for nearly a 100 years, clinicians still have a lot to learn. In recent years, a series of basic and clinical studies have shown potent protection for IHD by exogenous HEPs (15–19). These results have been confirmed in our laboratories (16, 20, 21).

Previous reviews focused either on the cellular and molecular mechanisms of HEPs which is too complex for clinical application (14, 22), or on presenting the clinical evidence which in turn is too simple for clinicians to understand their pathophysiological and pharmacological effects (15, 16). The purpose of this article is to bridge the gap between clinical and basic research.

## Overview of High-Energy Phosphates and Their Transformation

**Figure 1:**
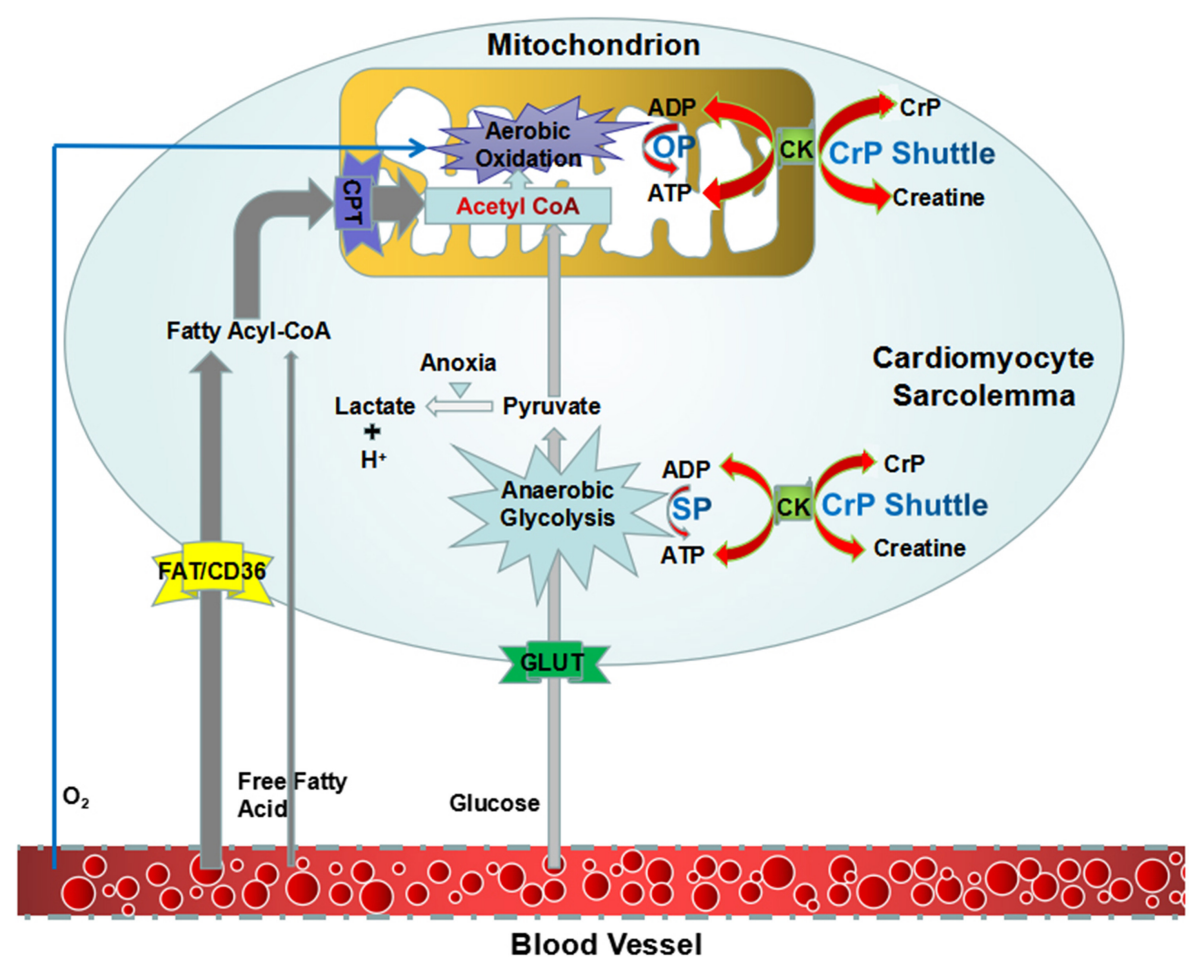
An overview of synthesis of ATP and “CrP shuttle” in cardiomyocyte. ATP is the intracellular energy currency, majority of which is synthesized from ADP by oxidative phosphorylation of mitochondria (predominant) and cytoplasmic substrate phosphorylation (subordinate). CrP is the storage and transport carrier of energy, which serves to transfer the HEP-bond from the site of ATP production to the site of ATP utilization through “CrP shuttle.” ADP, adenosine diphosphate; ATP, adenosine triphosphate; CK, creatine kinase; CrP, creatine phosphate; HEP, high-energy phosphate; OP, oxidative phosphorylation; SP, substrate phosphorylation.

It is believed that energy would be concentrated in the chemical bond containing phosphate groups, which yields energy upon hydrolysis (23). Low-energy phosphates are usually linked to phosphoester bonds, which will release 2 and 3 kcal/mol energy. HEPs include a variety of phosphate compounds with energies of hydrolysis higher than 7 kcal/mol (24). ATP and CrP are considered to be the primary HEPs in human body. ATP is the intracellular energy currency, majority of which is not synthesized *de novo* but generated from adenosine diphosphate (ADP) by oxidative phosphorylation (OP) of mitochondria and cytoplasmic substrate phosphorylation (SP) (Figure 1) (25, 26). Thus, at any given time, the total amount of ATP and ADP remains fairly constant and recycled continuously (27). While, CrP is the storage and transport carrier of energy, which serves to transfer the HEP-bond from the site of ATP production to the site of ATP utilization through “CrP shuttle” (Figure 1) (28–35). Normally the total quantity of ATP in human body is about 0.1 mole (~50 g). However, the energy used by human cells requires the hydrolysis of 100–150 moles (around 50–75 kg) of ATP daily (36). This means that each ATP molecule is recycled 1,000–1,500 times during a single day. The ATP and CrP activity combined, also referred to as the phosphagen system, is the most rapidly available source of energy (37). Unfortunately, the energy available from the store of phosphagen system is limited and can provide energy for a few seconds of maximal activity.

**Figure 2:**
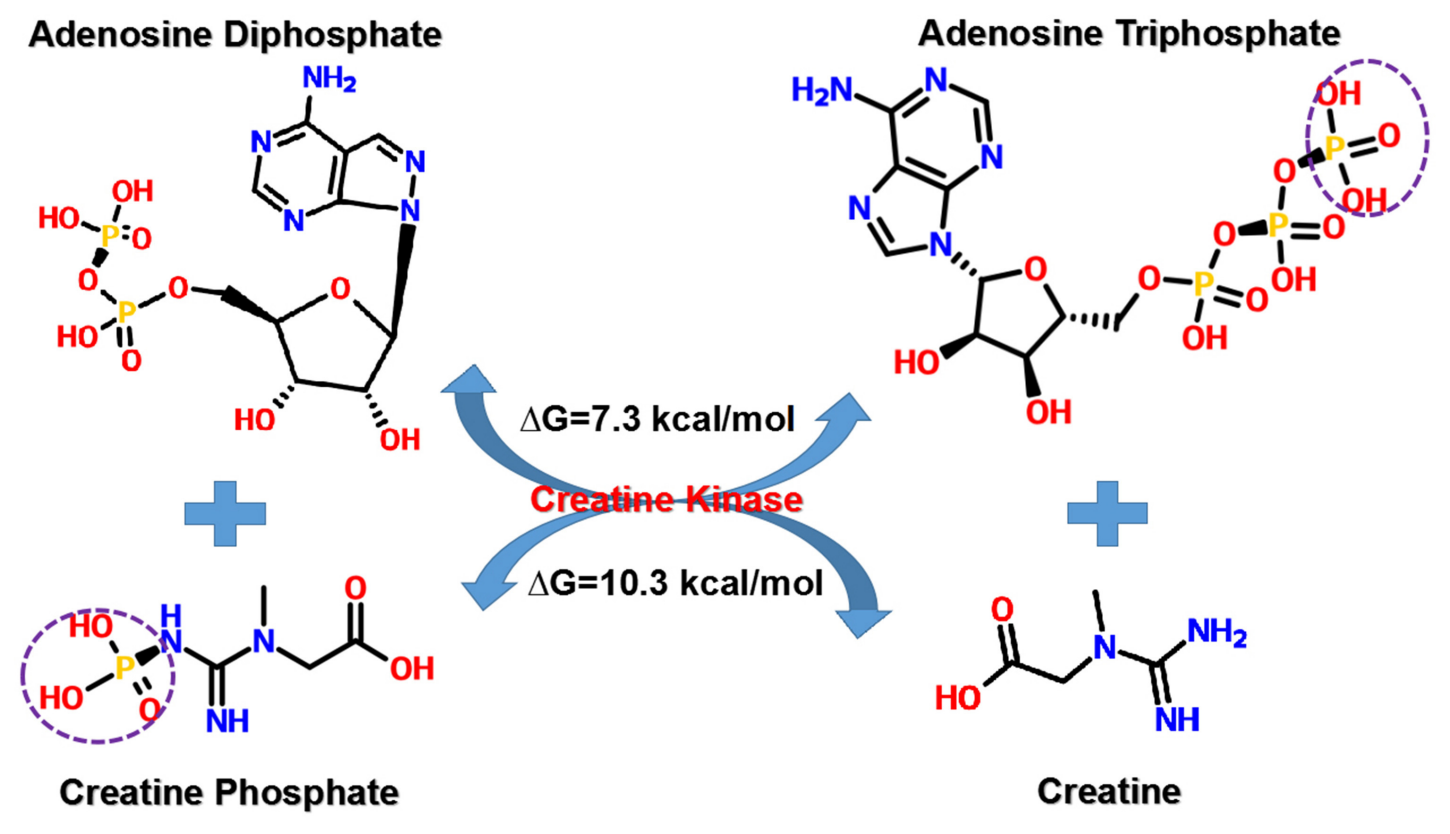
Transfer of HEP-bond through “CrP shuttle.” There is one HEP-bond (N~P) in the chemical structure of CrP. As compared, ATP has a relatively more complex molecular structure and two HEP-bonds (O~P). However, the N~P bond of CrP has more energy than either one O~P bond of ATP, 10.3 kcal/mol in comparison with 7.3 kcal/mol. ATP, adenosine triphosphate; CrP, creatine phosphate; HEP, high-energy phosphate; ΔG, Gibbs free energy change.

CrP, also known as phosphocreatine or phosphorylated creatine, is a small molecular compound with the formula of C_4_H_10_N_3_O_5_P, having a molecular weight of 211 daltons. There is one high-energy phosphate bond (N~P) in the chemical structure. As compared, ATP has a relatively more complex molecular structure (C_10_H_16_N_5_O_13_P_3_), larger molecular weight (507 daltons), and two high-energy phosphate bonds (O~P). However, the N~P bond of CrP has more energy than either one O~P bond of ATP, 10.3 kcal/mol in comparison with 7.3 kcal/mol (Figure 2) (38). Therefore, CrP can easily provide enough energy and serve as a HEP-bond donor for ATP reconstitution through “CrP shuttle” (28).

The contents of HEPs vary significantly in different tissues. The highest levels of HEPs are found in muscle, heart, brain, spermatozoa, and retina (14). The concentration and distribution of HEPs *in vivo* can be determined non-invasively by 31^P^-magnetic resonance spectroscopy (MRS) (39, 40). The myocardial CrP/ATP ratio measured by 31^P^-MRS reflects the viability and energy metabolic status of cardiomyocytes (41). Over a wide range of cardiac workloads, the CrP/ATP ratio is essentially invariant and consistent with a constant free ADP concentration (42, 43). The cutoff point for CrP/ATP ratio (>1.60 and <1.60), which was established retrospectively and need to be evaluated prospectively, is a stronger predictor of cardiovascular death (44). The ratio is decreased upon myocardial ischemia (45, 46).

## The Biosynthesis, Degradation and Turnover of Endogenous Creatine Phosphate

**Figure 3:**
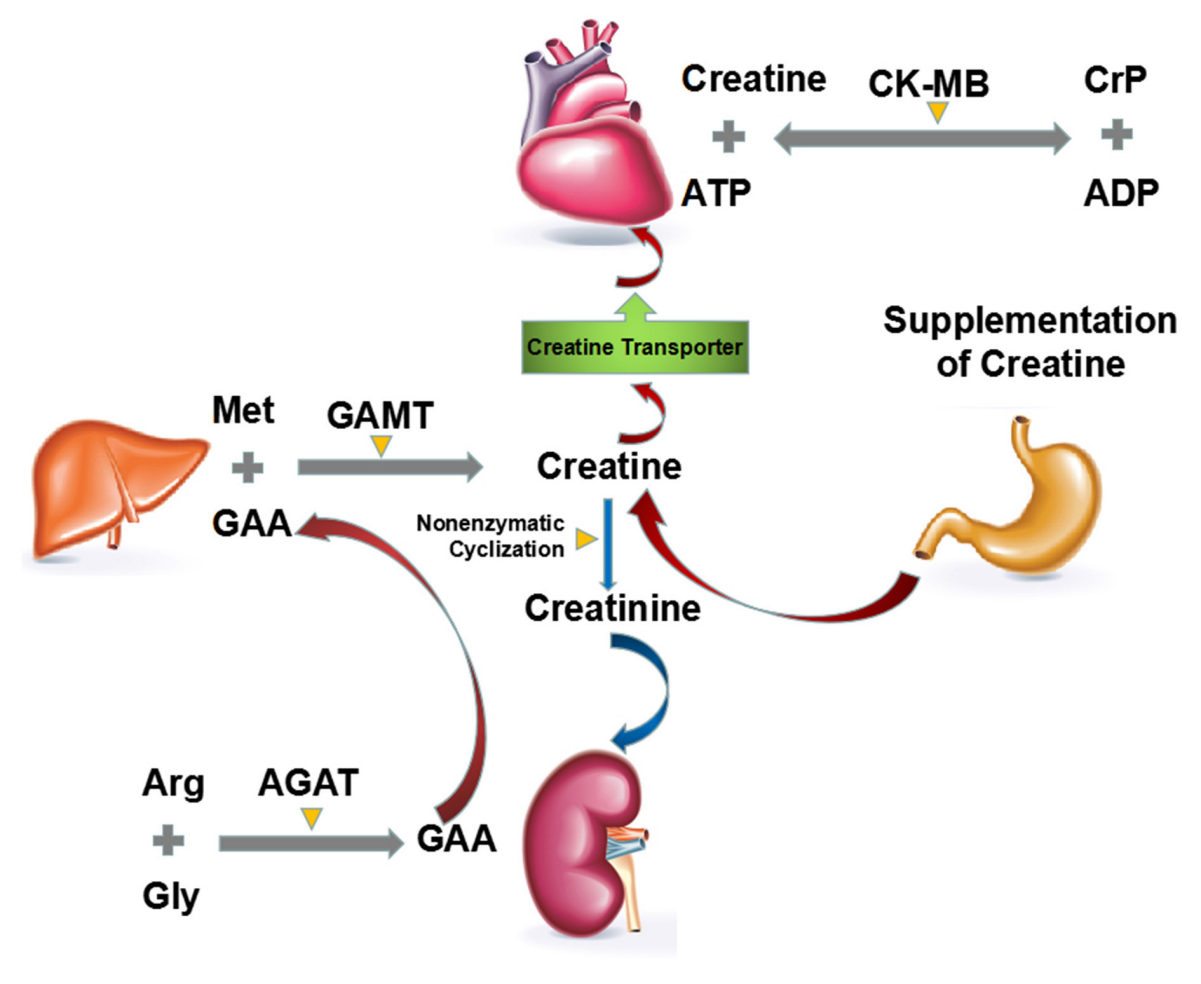
Major routes of biosynthesis, degradation and turnover of endogenous CrP. The biosynthesis of CrP begins by formation of creatine from three essential amino acids: arginine, glycine, and methionine. The main route of creatine synthesis involves formation of GAA in kidney, and methylation in liver. These reactions are, respectively, catalyzed by AGAT and GAMT. Then creatine is transported to heart by a specific Na^+^- and Cl^−^-dependent plasma membrane transporter. The degradation of creatine and CrP is an irreversible, non-enzymatic cyclization to creatinine, which should be supplemented by diet or *de novo* biosynthesis. ADP, adenosine diphosphate; AGAT, L-arginine:glycine amidinotransferase; ATP, adenosine triphosphate; CK, creatine kinase; CrP, creatine phosphate; GAA, guanidinoacetic acid; GAMT, S-adenosyl-L-methionine:N-guanidinoacetate methyltransferase.

The biosynthesis of CrP begins by formation of creatine from three essential amino acids: arginine, glycine, and methionine (Figure 3) (14). The entire glycine molecule is incorporated whereas arginine furnishes its amidino group to yield guanidinoacetic acid (GAA), which then methylated at the amidino group to give creatine. It is postulated, but largely accepted, that the main route of creatine synthesis involves formation of guanidinoacetate in kidney, and methylation in liver (47–49). These reactions are respectively catalyzed by two rate-limiting enzymes, i.e., L-arginine:glycine amidinotransferase (AGAT) and S-adenosyl-L-methionine:N-guanidinoacetate methyltransferase (GAMT) (47–50). To complete the phosphorylation process, creatine is then transported to tissues such as muscle, heart, and brain by a specific Na^+^- and Cl^−^-dependent plasma membrane transporter (51). CrP production is catalyzed by creatine kinase (CK), which is a dimer of M and B (M = muscle, B = brain) subunits produced by different structural genes. Three isozymes are possible: BB, MB, and MM. Cardiac muscle contains significant amounts of CK-MB (25–46% of total CK activity, as opposed to <5% in skeletal muscle), so that in myocardial infarction the rise in serum total CK activity is accompanied by a parallel rise in that of CK-MB (14, 52, 53).

Unlike the biosynthesis, the degradation of creatine and CrP is an irreversible, non-enzymatic cyclization to creatinine (Figure 3) (54, 55). Almost constant fraction of the body creatine (1.1%/day) and CrP (2.6%/day) is converted into creatinine, giving an overall conversion rate for total creatine pool (creatine + CrP) of ~1.7%/day (56).

For example, in a 70 kg man containing around 120 g of creatine pool, roughly 2 g/day are converted into creatinine and have to be replaced by creatine or CrP supplementation or from *de novo* biosynthesis (14).

## Myocardial Metabolic Changes During Ischemia/Reperfusion: Substrates, Pathways, Metabolites, and Purine Nucleotide Cycle

**Figure 4:**
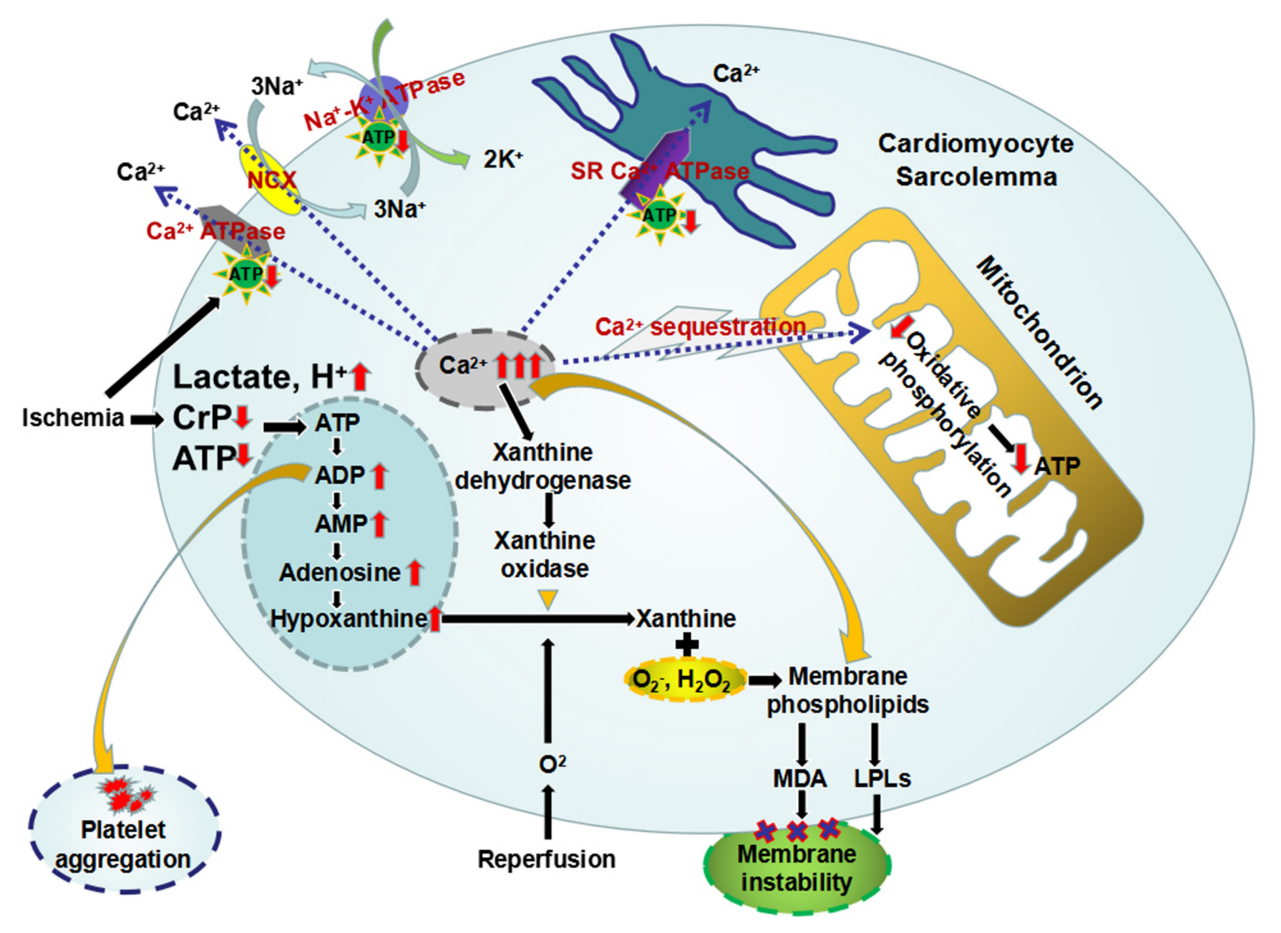
The primary metabolic changes and the secondary celluar injuries during myocardial ischemia/reperfusion. Ischemic myocardium preferentially utilizes the energy contained in CrP, followed by ATP, ADP, and AMP. And AMP can be further decomposed into adenosine and hypoxanthine, which leads to a decrease in intracellular adenine nucleotide pool. Furthermore, the lactic acid produced by glycolysis accumulate in cardiomyocytes, resulting intracellular acidosis. The loss of HEPs eliminates three of the four mechanisms of cellular calcium homeostasis, leading intracellular Ca^2+^ overload. Mitochondrial sequestration, the remaining mechanism, causes overloading of the mitochondria with Ca^2+^ and diminished capacity for oxidative phosphorylation. And overloaded intracellular Ca^2+^ induces the conversion of xanthine dehydrogenase to xanthine oxidase. The latter can produce oxygen free radicals, which in turn oxidize the membrane phospholipids and produce MDA, causing the membrane instability. In addition, intracellular accumulation of metabolic intermediates, including AMP, lactic acid, Ca^2+^, and H^+^, etc, may activate membrane phospholipase to make cell membrane degrade to LPLs, which also contribute to myocardial membrane instability. Increased ADP can induce platelet adhesion and aggregation. ADP, adenosine diphosphate; AMP, adenosine monophosphate; ATP, adenosine triphosphate; CrP, creatine phosphate; HEPs, high-energy phosphates; LPLs, lysophospholipids; MDA, malondialdehyde.

Within a few seconds after coronary blood flow blockage, the oxygenated hemoglobin in ischemic zone rapidly depletes. The main pathway used to generate energy in myocardium changes from aerobic oxidation of mitochondria to cytoplasmic anaerobic glycolysis (Table 1) (57, 58). And the primary substrate of myocardial energy metabolism also changes from free fatty acids to glucose (Table 1) (57–61). However, the HEPs synthesized by glycolysis are far from meeting the energy requirements of heart. Under such condition, the ischemic myocardium preferentially utilizes the energy contained in endogenous CrP, followed by ATP, ADP, and adenosine monophosphate (AMP) (Figure 4) (62–67). AMP can also be decomposed into adenosine, hypoxanthine, etc. under the action of 5'-nucleotidase (Figure 4) (62, 68). The above reaction ultimately leads to a decrease in intracellular adenine nucleotide pool (ATP + ADP + AMP), resulting in a significant reduction in high-energy phosphate precursors. If the myocardium recover aerobic oxidation in a short period of time, AMP can be reoxidized to ADP and ATP to replenish energy. If not, it is no longer possible to reoxidize AMP to ADP or ATP. Furthermore, the lactic acid and other intermediate products produced by glycolysis accumulate in cardiomyocytes (Figure 4) (57, 58). After 10 min of ischemia, the intracellular pH will drop to 5.8–6.0 (69, 70). The rate of ADP rephosphorylation to ATP by anaerobic glycolysis is slowed down by acidosis (71).

**Table 1:** Myocardial energy metabolism: source, process and site of ATP production.

**Source of ATP production**	**Pathway of ATP production**	**Oxygen consumption (per unit ATP)**	**Accumulation of acid metabolites**	**Rate of ATP production**	**Net ATP yield (per unit substrate)**	**Site of ATP production**
CrP	CrP ⇆ ATP shuttle	None	–	Very fast	1	Cytoplasm
Glucose	Anaerobic glycosis	None	+ + +	Fast	2	Cytoplasm
Glucose	Aerobic oxidation	Less	–	Moderate	38	Mitochondria (predominant) and cytoplasm
Free fatty acids	Aerobic oxidation	More	–	Slow	Usually > 100 (depending on the number of carbon atoms in the molecule of free fatty acid)	Mitochondria (predominant) and cytoplasm

*ATP, adenosine triphosphate; CrP, creatine phosphate/*.

Secondary to the metabolic changes, myocardial ischemia/reperfusion injuries occur as follows: intracellular Ca^2+^ overload, accumulation of arrhythmogenic intermediates and oxygen free radicals, myocardial membrane instability, electrophysiological changes in cardiomyocytes, mitochondrial damage, and platelet aggregation, etc (Figure 4).

## Pathophysiological and Pharmacological Effects of Exogenous Creatine Phosphate on Myocardial Ischemia

The clinical effects of ATP in patients with cardiovascular disorders have been evaluated in early studies (72–74). Intravenous administration of ATP can interrupt the reentry pathways through the atrial ventricular node and restore normal sinus rhythm accompanied by relatively high incidences of advanced atrioventricular block and other adverse reactions, which makes paroxysmal supraventricular tachycardia the primary cardiovascular indication (75). And it seems quite paradoxical that oral administration of ATP may lead to a progressive diminution of plasma ATP level (76). Furthermore, exogenous ATP is a charged molecule containing three negative charges that is not freely permeable through cell membranes (77–79). In addition, there are enzymes that decompose ATP on the surface of cell membrane, including ATPase, adenylate kinase and AMP deaminase, which can split ATP into ADP, AMP, adenosine, and inorganic phosphate (80, 81). Since the first publication by Parrat and Marshall (82), CrP has been substantially demonstrated to be effective in protection of ischemic myocardium. The following we will focus on the pathophysiological and pharmacological effects of exogenous CrP, including but not limited to supplementing cellular energy.

### Replenishment of Intracelluar ATP

It has been observed that the exogenous CrP could be incorporated into intracellular ATP molecules and increase the tissue level of ATP (83). Although exogenous CrP uptake was 3–4 orders of magnitude lower than ATP conversion in the case of normal cardiac work, it may be important in maintaining subsarcolemmal pools of CrP or ATP (Figure 4) (35, 83). The exogenous CrP uptake rate can be markedly increased in hypokinetic segments of ischemic myocardium (35, 83–86). Low-dose CrP may promote intracellular ATP synthesis mainly through substrate. After reaching a certain concentration of 10 mmol/L, it can significantly inhibit 5'-nucleotidase and AMP deaminase, thereby maintaining the nucleotide pool level, indicating that CrP does not only act as a energy substrate but also a regulator able to bind to the active sites of the enzymes and change their activity (62, 68, 87–90).

### Attenuation of Intracellular Ca^2+^ Overload in Cardiomyocytes

Normally, extracellular fluid has a concentration of Ca^2+^ 10,000 times higher than intracellular fluid (91). Furthermore, there is an electrical force driving Ca^2+^ into the cell because of the negative resting membrane potential (91, 92). However, there is little leakage of Ca^2+^ into the cardiomyocyte except during the action potential. Even the Ca^2+^ that enters the cell during action potentials must be removed from the cell otherwise an accumulation of Ca^2+^ would lead to cellular dysfunction (92). Main mechanisms maintaining the intracellular to extracellular concentration and charge gradients include: (1) pumping Ca^2+^ out of the cytoplasm by the plasma membrane Ca^2+^ ATPase (93), (2) exchange of Ca^2+^ for Na^+^ driven by the intracellular to extracellular concentration gradient of Na^+^ as a result of the plasma membrane Na^+^-K^+^ ATPase (94), (3) sequestration of cytoplasm Ca^2+^ in sarcoplasmic reticulum (SR) by the SR Ca^2+^ ATPase (95), and (4) accumulation of intracellular Ca^2+^ by oxidation-dependent calcium sequestration inside the mitochondria (96). The loss of HEPs during ischemia eliminates three of the four mechanisms of cellular calcium homeostasis (Figure 4). Mitochondrial sequestration, the remaining mechanism, causes overloading of the mitochondria with Ca^2+^ and diminished capacity for oxidative phosphorylation (Figure 4) (97). Furthermore, activation of phospholipases and protein kinases (98), production of arachidonic acid (99, 100), and oxygen free radicals (101) are all involved in the destruction of membrane integrity. This, in turn, causes a massive and rapid influx of Ca^2+^ into the cell.

Several studies have shown that intracellular Ca^2+^ overload is a major cause of myocardial cell damage and cardiac dysfunction in IHD. CrP can reduce Ca^2+^ influx by providing energy to ATP-dependent Ca^2+^ ATPase and Na^+^-K^+^ ATPase on the plasma membrane (102, 103). At the same time, the Ca^2+^ ATPase activity on the sarcoplasmic reticulum is restored, and Ca^2+^ enter the sarcoplasmic reticulum to avoid the myocardial stiffness contracture (104). Furthermore, CrP binds to membrane phospholipids through zwitterionic interaction, which can enhance membrane stability (105, 106). In addition, CrP can also provide energy for the sliding of actin-myosin filaments, promoting the rapid recovery of myocardial contractility (107).

### Protection of Heart From Oxidative Stress-Induced Myocardial Injury

And overloaded intracellular Ca^2+^ induces the conversion of xanthine dehydrogenase to xanthine oxidase (108–111). The latter can produce superoxide and xanthine from hypoxanthine upon reperfusion (Figure 4) (112). Furthermore, more damaging free radicals could be produced by the metal catalyzed Haber-Weiss reaction (113–115). The large amount of oxygen free radicals generated by the above reactions can in turn oxidize the membrane phospholipids and produce malondialdehyde (MDA), causing the membrane instability (Figure 4) (116). Zucchi et al. (117) found that supplementation of exogenous CrP could reduce the product of phospholipid peroxidation, MDA, by inhibiting ADP/AMP degradation and Ca^2+^ accumulation in cardiomyocytes. Myocardial peroxidation damage is alleviated through all of the above mechanisms.

### Stabilization of Membrane Structure

Maintaining the integrity of the phospholipid bilayer membrane is a basic requirement for preserving overall cell viability. Myocardial membrane instability due to the decrease of ATP production and accumulation of acid metabolites plays a key role in the pathogenesis of ischemia-reperfusion injury, especially the electrophysiological manifestation of ischemia (118). The possibility that lysophospholipids (LPLs) contribute to myocardial membrane instability was first reported by Hajdu (119). Normally their concentration is maintained very low, but LPLs in sufficient quantities are potent detergents, which can alter general properties of the membrane such as fluidity and permeability (120). Furthermore, LPLs have been shown to affect the activities of plasma membrane Na^+^-K^+^ ATPase (121). Upon myocardial ischemia, intracellular accumulation of metabolic intermediates, including AMP, lactic acid, Ca^2+^, and H^+^, etc, may activate membrane phospholipase to make cell membrane degrade to LPLs (Figure 4). At 8 min after ischemia, a 60% increase in LPLs levels occurred, which could either be reacylated or transacylated to form precursor phospholipids or further degraded, depending on the energy state of the cell (121–123). Supplementation of exogenous CrP can provide energy to ATP-dependent Ca^2+^ ATPase and Na^+^-K^+^ ATPase on the plasma membrane and reduce the activation of anaerobic glycolysis, which blocks the process of phospholipids degradation and stabilizes the cell membrane. In addition, the integrity of the mitochondrial structure during ischemia is the basis for oxidative phosphorylation to synthesize ATP after reperfusion. CrP also has protective effects on the mitochondrial membrane and its oxidative phosphorylation function (124–126).

### Broad Spectrum Antiarrhythmic Effects

Normally, the electrophysiological properties of cardiomyocytes require cell membrane integrity and maintaining of intracellular to extracellular concentration and charge gradients. Metabolic changes after myocardial ischemia, including the decrease of ATP production and accumulation of acid metabolites, lead to decreased activity of ATP-dependent transport systems. ATP-sensitive K^+^ channels (KATP), inactivated by normal cellular ATP levels, will open and permit K^+^ to leave the cell upon ischemia (127, 128). Furthermore, decreased activity of Na^+^/K^+^-ATPase leads to extracellular accumulation of K^+^ and inactivation of fast Na^+^ channels that are responsible for the rapid depolarization (129). These mechanisms lead to a series of electrophysiological changes in cardiomyocytes, including: (1) the resting membrane potential and the action potential amplitude are significantly decreased; (2) the depolarization speed is slowed down; (3) the action potential duration (APD) is shortened; (4) the distance from the resting membrane potential to the K^+^ equilibrium potential is increased; (5) the conduction velocity rate is slowed down (130). All of the above changes ultimately can contribute to arrhythmias.

Studies have shown that in myocardial ischemia and reperfusion, CrP can play a broad spectrum antiarrhythmic effects through several electrophysiological mechanisms, including but not limited to ATP replenishment (131). Firstly, by providing energy to ATP-dependent KATP channels and Na^+^/K^+^-ATPase, exogenous CrP can reduce extracellular accumulation of K^+^ and reactivate the fast Na^+^ channels, suggesting a Class I antiarrhythmic role (132). Secondly, by prolonging ventricular myocardium APD and effective refractory period (ERP) under normoxic but not ischemic conditions, exogenous CrP can prevent reentrant circuits forming between the ischemic and non-ischemic zone and play a class III antiarrhythmic role (132, 133). Thirdly, by attenuating intracellular Ca^2+^ overload, exogenous CrP can inhibit Ca^2+^-mediated activation of inward current channels and triggered activity, exerting a class IV antiarrhythmic role (134, 135). Furthermore, exogenous CrP can also play an antiarrhythmic role by reducing the accumulation of arrhythmogenic lysophosphoglycerides and increasing the threshold of ventricular fibrillation (136–138).

### Inhibiting Platelet Aggregation and Improving Microvascular Function

It is known that ADP can not only induce platelet adhesion and aggregation, but also amplify the aggregation effects of collagen, thrombin and other inducers (Figure 4) (139, 140). ADP may still affect the platelets when the arachidonate pathway is blocked (141). Exogenous CrP can inhibit platelet aggregation and then improve the microvascular function by rapid removal of ADP and formation of ATP, which is an inhibitor of ADP-induced platelet aggregation (19, 142).

## Clinical Application of Exogenous Creatine Phosphate in Ischemic Heart Disease: Evidence and Evaluation

As mentioned above, energy metabolic abnormalities are the upstream and primary pathophysiologic manifestation of myocardial ischemia. Whereas, hemodynamic, electrophysiological, morphological, clinical, biochemical and imaging changes are the downstream, and secondary consequence of myocardial energy metabolic abnormalities. The depletion of HEPs is involved in both upstream and downstream changes in myocardial ischemia. As demonstrated *in vitro* and animal experiments, CrP was suggested to be potentially beneficial in patients with acute and chronic myocardial ischaemic injury through multiple mechanisms, including but not limited to ATP replenishment. In fact, results from a large number of clinical studies substantially support that supplementation of exogenous CrP is associated with improved short-term survival (143, 144), enhancement of cardiac systolic and diastolic function (145–147), lower peak CK-MB/troponin release (20, 148–152), reduction in the incidence of major arrhythmias (144, 151, 153–156), etc. There is still uncertainty, however, whether the administration of exogenous CrP can improve long-term outcomes, rather than just the secondary endpoints or pathophysiological process of IHD.

## Limitations and Perspectives

According to a meta-analysis performed by Landoni et al. (16), although more than 4,000 articles were screened, only 12 studies comparing CrP with placebo or standard treatment in patients with IHD met the design requirements for controlled or case-matched clinical trials. Unfortunately, there is insufficient statistical power to obtain results on long-term survival due to the common limitations, including: (1) single center trial; (2) small sample size; (3) short-term follow-up; (4) secondary end-points; (5) choice of standard treatment rather than placebo as the comparator; (6) administration routes and doses of CrP varying significantly among the studies; (7) inadequate baseline information or baseline bias (20, 143, 144, 146, 150, 151, 153, 156). In addition, majority of the studies were published before the “era of revascularization” and patients were recruited from those undergoing non-revascularization therapy or mixed, significantly different from the current practice (143, 144, 146, 150, 153, 156).

At first glance, it is surprising that exogenous CrP has not been shown to improve long-term survival in clinical studies. In fact, there are two sides to the same issue. On one side, CrP may plays extensive roles in every physiological and pathophysiological process from upstream to downstream of myocardial ischemia. On the other side, the myocardial intracellular actions of CrP lack target and pathway specificity. Furthermore, the uptake and distribution of exogenous CrP *in vivo* lack of tissue and cell specificity. Such non-specificities lead to uncertainties in the dominant pharmacological mechanism, optimal administration route and dose, as well as treatment window of exogenous CrP in individualized patients with IHD. Moreover, the cardioprotection of exogenous CrP may be limited by endogenous CrP levels. However, owing to the physiological, pathophysiological, and pharmacological plausibility of its effects and to the concordance of the beneficial effects of exogenous CrP on multiple secondary but important outcomes and short-term survival, there is urgent need for high-quality multicentre randomized controlled trials (RCTs) to confirm long-term survival improvement. In addition, further studies are needed to investigate the causality between changes in endogenous/exogenous CrP levels and IHD progression and prognosis (157).

To better understand the pathophysiological and pharmacological effects, we specified the context for all cited researches as cell study (19, 23–27, 29–35, 48–54, 69, 70, 91–103, 118, 128), animal study (12, 41, 42, 45, 46, 65, 66, 68, 71–74, 76–78, 81–90, 99, 104, 111, 116–119, 129, 133–135) and human study (15–18, 20, 21, 37–40, 44, 58, 108–110, 143–156). Furthermore, we detailed the indications, contraindications, side effects, and application instructions of CrP supplement in Table 2.

**Table 2:** The indications, contraindications, side effects, and application instructions of CrP supplement for IHD.

**Indications**	**Contraindications and relative contraindication**	**Side effects**	**Instructions of administration and dosage**
• Cardiac metabolic abnormalities during myocardial ischemia. • Cardioprotection during heart surgery.	• Chronic renal failure (in high doses, for example, daily dose of 5–10 g). • Hypersensitivity to drug components. • Pregnancy.	• Allergic reactions. • Lowering of arterial pressure.	• Cardiac metabolic abnormalities during myocardial ischemia: 0–24 h—intravenous bystry infusion of 2–4 g of CrP divorced in water for injections of 50 ml with the subsequent intravenous infusion for 2 h 8–16 g in 250 ml of 5% of solution of glucose; during second day 2 times a day intravenously kapelno (infusion duration of 30 min) enter 2–4 g of the drug divorced in 50 ml of water for injections; during third day the drug is administered according to the same scheme in a dose 2 g (if necessary treatment is continued for 6 days). • Cardioprotection during heart surgery: intravenously kapelno (infusion duration of 30 min) 2 g of the drug divorced in 50 ml of water for injections with frequency rate of introduction 2 times a day. The course is begun in 3–5 days prior to surgical intervention and continued 1–2 more days after its carrying out. During operation it is necessary to add to composition of usual cardioplegic solution in concentration 10 mmol/l just before introduction.

*CrP, creatine phosphate; IHD, ischemic heart disease*.

## Conclusions

The purpose of this article is to provide a comprehensive and concise description of the cellular and molecular aspects of cardioprotective mechanisms and a critical evaluation of the clinical evidence of HEPs in IHD. According to the well-documented physiological, pathophysiological and pharmacological properties of HEPs, exogenous CrP may be considered as an ideal metabolic regulator. It plays cardioprotection roles from upstream to downstream of myocardial ischemia through multiple complex mechanisms, including but not limited to replenishment of cellular energy. Although exogenous CrP administration has not been shown to improve long-term survival, the beneficial effects on multiple secondary but important outcomes and short-term survival are concordant with its pathophysiological and pharmacological effects. There is urgent need for high-quality multicentre RCTs to confirm long-term survival improvement in the future.

## Author Contributions

HY-D, ZY-X, and YS-W contributed toward drafting and critically reviewing the document and agree to be accountable for all aspects of the work. YS-W and ZY-J provided his views and comments on the manuscript, made the final decision about the journal selection as well as approved the submission of the manuscript to the journal. All authors contributed to the article and approved the submitted version.

## Conflict of Interest

The authors declare that the research was conducted in the absence of any commercial or financial relationships that could be construed as a potential conflict of interest.

## Publisher's Note

All claims expressed in this article are solely those of the authors and do not necessarily represent those of their affiliated organizations, or those of the publisher, the editors and the reviewers. Any product that may be evaluated in this article, or claim that may be made by its manufacturer, is not guaranteed or endorsed by the publisher.

## Abbreviations

ADP: adenosine diphosphate
AGAT: L-arginine:glycine amidinotransferase
AMP: adenosine monophosphate
APD: action potential duration
ATP: adenosine triphosphate
CK: creatine kinase
CrP: creatine phosphate
ERP: effective refractory period
GAA: guanidinoacetic acid
GAMT: S-adenosyl-L-methionine:N-guanidinoacetate methyltransferase
HEPs: high-energy phosphates
IHD: ischemic heart disease
KATP: ATP-sensitive K^+^ channels
LPLs: lysophospholipids
MDA: malondialdehyde
MEMT: myocardial energy metabolic therapy
MRS: magnetic resonance spectroscopy
OP: oxidative phosphorylation
RCTs: randomized controlled trials
SP: substrate phosphorylation.
